# Bladder’s Blind Spot: A Rare Case of Non-bilharzial Diverticular Squamous Cell Carcinoma Treated With Partial Cystectomy

**DOI:** 10.7759/cureus.81144

**Published:** 2025-03-25

**Authors:** Roshan Reddy, Rajan Ravichandran, Velmurugan Palaniyandi, Hariharasudhan Sekar, Sriram Krishnamoorthy

**Affiliations:** 1 Urology, Sri Ramachandra Institute of Higher Education and Research, Chennai, IND

**Keywords:** bladder diverticulum resection, bladder malignancy, non-bilharzial, partial cystectomy, squamous cell carcinoma

## Abstract

Squamous cell carcinoma (SCC) is an uncommon malignancy found within the bladder diverticulum. Early extravesical invasion is more likely to occur in diverticula when there is no muscle layer present. The gold standard for bladder SCC is radical cystectomy (RC), although in individuals with poor performance status, it might not be feasible. This case report describes a rare example of primary intra-diverticular SCC that was treated well with adjuvant radiation therapy and partial cystectomy (PC). A 68-year-old man was experiencing increasing frequency of urination and painless hematuria for three months. In a bladder diverticulum, moderately differentiated SCC (pT3aN0M0) was confirmed by imaging and histological examination. RC was not a viable alternative due to the poor performance condition. The patient received adjuvant radiation for microscopic extravesical extension after undergoing a PC and bilateral pelvic lymphadenectomy. During a five-year follow-up, routine cystoscopy and yearly imaging revealed that he was symptom-free and had not experienced any metastases or recurrence. This scenario shows that for certain individuals who are not suitable candidates for RC, PC combined with lymphadenectomy and adjuvant radiation therapy is a good substitute for localized bladder diverticular SCC. Due to SCC's aggressive nature and high recurrence rates, long-term surveillance, aggressive management, and early identification are crucial for bladder-preserving strategies.

## Introduction

Bladder cancer, the tenth most common and aggressive cancer globally, is typically sub-categorized as urothelial carcinoma (UC) and non-urothelial cancer with variable differentiation. The type of squamous cell carcinoma (SCC) is non-urothelial. Accounting for a prevalence of one to nine percent, bladder tumors discovered in the diverticulum are uncommon [1]. Of diverticular cancers, SCC makes up 20-25%, with urothelial malignancy accounting for the remaining portion. Chronic inflammation and urine stasis inside a weakly contractile diverticulum are the pathophysiological causes. In the bladder diverticulum, squamous metaplasia is fueled by persistent inflammation and urine stasis, which raises the possibility of malignant transformation. Genetic alterations, oxidative stress, and inflammatory mediators are all brought on by prolonged irritation, and urinary carcinogens hasten the development of tumors. Prolonged urine retention due to the absence of a muscle layer exacerbates chronic irritation and promotes the growth of SCC. Bladder SCC is an uncommon urological cancer that is more frequently linked to bilharziasis. The prevalence of non-bilharzial SCC (NB-SCC) in bladder cancer patients is just two to three percent [2]. Although the guidelines provide the best treatment for most bladder SCC, there are no particular guidelines for managing bladder diverticulum SCC. Radical cystectomy (RC) is considered the gold standard treatment for bladder SCC; however, it was not a viable option in this patient due to poor performance status. This article describes a unique instance of a primary intra-diverticular SCC that was successfully treated with partial cystectomy (PC) with great effect.

## Case presentation

A 68-year-old man who has had painless intermittent hematuria for three months visited the outpatient department. For a month, the patient complained of increased micturition frequency. There was no history of flank pain or fever. Fifteen years ago, he had a cystolithotripsy for a bladder stone. Despite not smoking, he had a history of hypertension and diabetes. Examination revealed a soft abdomen and no palpable bladder. According to blood analysis, hemoglobin was 9.7 g/dl (reference range for men: 13.2 to 16.6 g/dL) and creatinine was 2.4 mg/dl (reference range for men: 0.7 to 1.3 mg/dL (61.9 to 114.9 µmol/L)). A normal urine test showed higher levels of leukocytes and red blood cells. Samples of urine cultures came out negative. The diverticulum of the bladder had a hyperechoic lesion, according to an abdominal ultrasonography.

**Figure 1:**
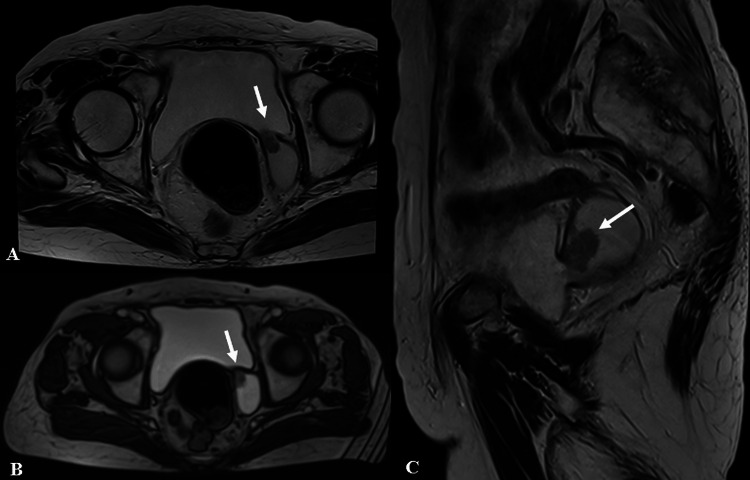
Radiographic finding: A 2.7 x 2.1 cm papillary lesion (white arrows) on magnetic resonance urography imaging of the left posterolateral bladder wall inside a diverticulum with intermediate signal intensity (A and B show the axial view while C shows the sagittal view).

During the cystoscopy, a diverticulum with a polypoidal growth was seen on the bladder's left lateral wall. A single intra-diverticular lesion, 2.7 x 2.1 cm in size, was seen in the left posterolateral bladder wall on magnetic resonance urography imaging (Figure 1). There is no hydroureteronephrosis visible. Only the superficial layer of bladder samples was removed from the growth and checked for histological analysis since the diverticulum lacks a muscular layer. A SCC tissue diagnosis was acquired. The patient was unfit for RC and had a poor performance status. Therefore, bilateral pelvic lymph node dissection and PC were performed.

**Figure 2:**
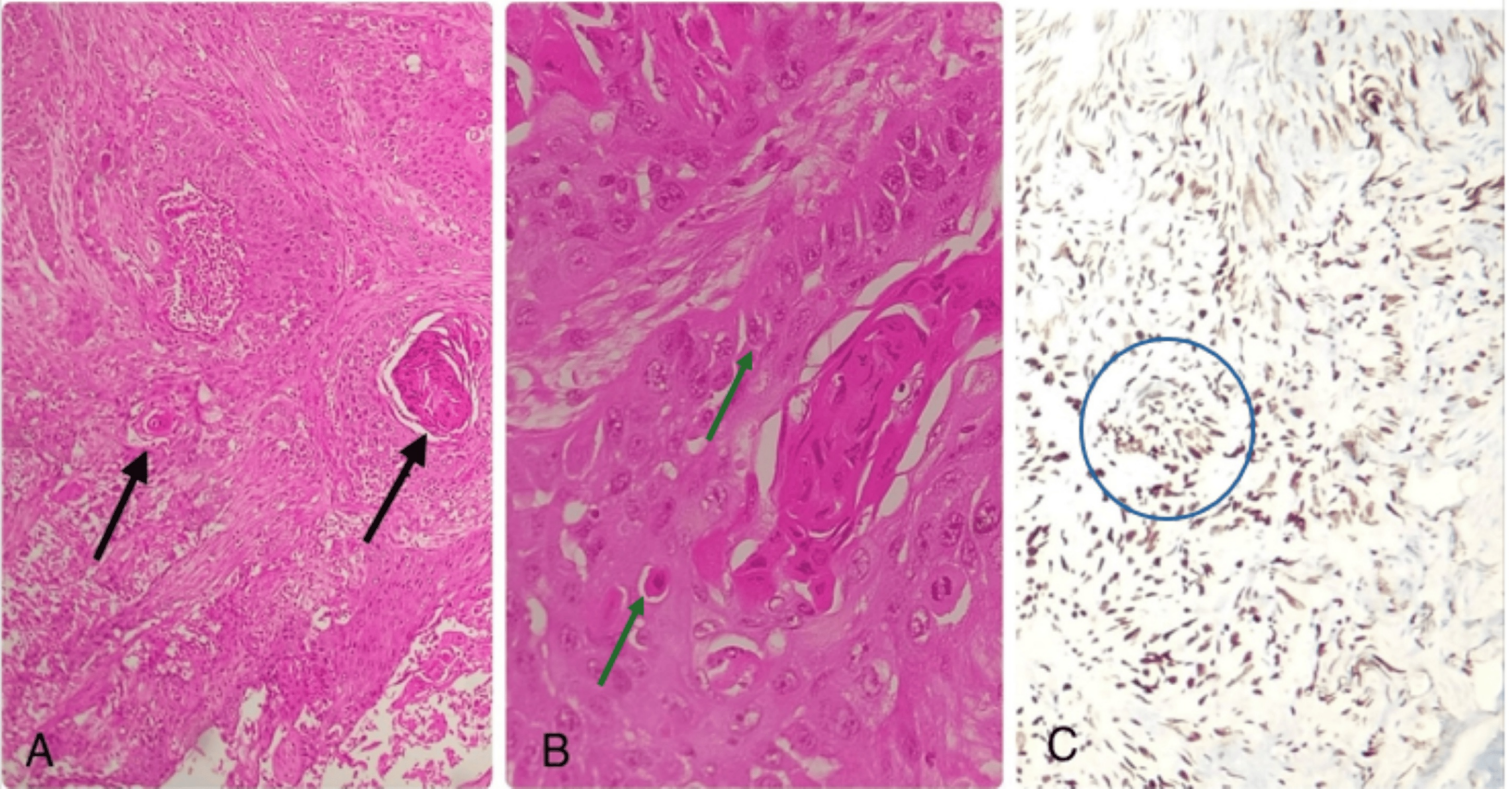
Bladder tumor histopathological analysis using immunohistochemistry (IHC) labeling. A: Hematoxylin and eosin stain 10x on squamous cells with keratin pearls (black arrows), B: Apoptotic keratinocytes and mitotic figures forming intercellular bridges (40x magnification of Hematoxylin & eosin stain) (green arrows), and C: IHC markers demonstrating P40 nucleus positivity (blue circle).

There were pelvic lymph nodes, a tumor lesion in the bladder diverticulum, and around 1-1.2 cm border of normal bladder wall in the removed specimen. The surgical margins were negative, and according to the histopathological evaluation, pT3aN0M0 was SCC of a moderately differentiated entity. Nucleus positivity for P40 was demonstrated by immunohistochemistry (IHC) (Figure 2). Histopathological examination demonstrated microscopic extravesical invasion into the perivesical fat but no involvement of lymph nodes. Ionizing radiation therapy was proposed to be an adjuvant treatment because of microscopic extravesical expansion. On the tenth day following surgery, the patient was discharged. Three months later, the patient was symptom-free. Regularly scheduled cystoscopies revealed no tumorous growths. Annual pelvic and abdominal computed tomography (CT) imaging revealed no signs of recurrence during the five-year follow-up.

## Discussion

About two to five percent of bladder cancer is NB-SCC, which is an uncommon and aggressive form of malignancy [3]. NB-SCC is caused by chronic irritative stimuli, including smoking, bladder calculus, injury to the spinal cord, prolonged catheterization, the presence of foreign bodies in the bladder, and recurrent urinary tract infections, in contrast to bilharzial SCC, which is closely linked to Schistosoma haematobium infection [4]. By upregulating inflammatory mediators and pro-angiogenic growth factors, these factors cause squamous metaplasia, dysplasia, and ultimately malignant transformation [5]. Although SCC often begins in the bladder's lateral wall and trigone, its low prevalence makes it uncommon to arise within a bladder diverticulum [6].

With painless hematuria being the most prevalent symptom, bladder SCC generally presents clinically insidiously, often delaying diagnosis [7]. Locally advanced stage (T3-T4b), lymph node involvement, and advanced age (>70 years) are prognostic markers for SCC [8]. Furthermore, hydroureteronephrosis, which indicates significant local illness and potential ureteric obstruction, is acknowledged as an independent poor prognostic factor [9].

Bladder SCC requires a vigorous and early surgical approach because, in contrast to urothelial cancer, they respond poorly to systemic chemotherapy [10]. The gold standard for muscle-invasive SCC is still RC with urinary diversion, especially for individuals with operable disease [11]. However, in extremely rare circumstances, bladder-preserving techniques, such as PC, might be considered. A single tumor away from the vesicoureteral junction, at least 2 cm negative surgical margins, no carcinoma in situ, and full transurethral resection before surgery are among the strict criteria for PC that the Memorial Sloan Kettering Cancer Centre has proposed [12].

Golijan et al. recommended partial or radical cystectomy for T3+ tumors and categorized bladder diverticular tumors as superficial (Ta, Tis), superficially invasive intra-diverticular (T1), and extra diverticular invasive (T3a, T3b) lesions [7]. In their comparison of radical and partial cystectomy, Capitanio et al. showed that, in carefully chosen cases, PC provides similar overall and disease-free survival rates [9]. Like this, Yin et al. documented a PC-treated localized SCC with a 10-year disease-free survival, suggesting the potential use of this strategy in carefully chosen patients [10].

The patient in the current instance had bladder diverticulum-confined T3aN0M0 SCC. In cases with localized SCC, the presence of microscopic extravesical extension (pT3a) without nodal involvement (pN0) emphasizes the significance of comprehensive surgical resection and adjuvant radiotherapy. This is consistent with other observations that show the lack of a muscle layer in diverticular SCC can lead to early extravesical dissemination, highlighting the importance of close postoperative monitoring. A different strategy was required because RC was not recommended due to his low-performance condition. To maximize local disease management, a PC and bilateral pelvic lymphadenectomy were carried out, followed by adjuvant radiation therapy. The patient showed no evidence of recurrence or metastasis during the five-year follow-up, confirming the viability of this strategy in certain patients. Although RC is still the recommended treatment for SCC, PC may be a good substitute in carefully chosen cases, especially for patients who are not candidates for radical surgery, as seen by the success of PC in this instance.

We compared the treatment results of bladder diverticular SCC instances to put our findings into context (Table 1). Although RC is still the gold standard of treatment, certain cases treated with adjuvant therapy and PC have demonstrated similar long-term disease control. Our example demonstrates that bladder-preserving techniques are feasible for patients who are appropriately selected.

**Table 1:** Comparative outcomes of bladder diverticular SCC treatments SCC: Squamous cell carcinoma

Study	Diagnosis	Patients (n)	Treatment modality	Tumor stage (pTNM)	Survival/recurrence outcome	Follow-up duration
Golijanin et al. (2003) [7]	Bladder diverticular SCC	12	Radical cystectomy (RC)	T3a-T4a	5-year DFS: 48%	5 years
Štimac et al. (2015) [13]	Bladder diverticular SCC	1	Bladder-sparing surgery	T2N0M0	5-year DFS: not assessed	3 years
Current case	Bladder Diverticular SCC	1	Partial cystectomy + Radiation	T3aN0M0	5-year DFS: 100%	5 years

Despite these encouraging results, a significant drawback is still the dearth of extensive prospective trials assessing PC outcomes in bladder SCC, especially when diverticular tumors are present. To confirm the effectiveness of PC in this context, bigger patient cohorts and longer follow-up studies are required, considering the aggressive character and high recurrence rate of bladder SCC.

Learning points

The high recurrence incidence of bladder SCC makes periodic post-treatment surveillance using imaging and cystoscopy necessary. Patients with bladder diverticula are more likely to experience early extravesical tumor invasion because they lack a muscular layer. Moreover, hydroureteronephrosis is acknowledged as an independent indicator linked to an adverse outcome. PC has become a feasible option in certain carefully chosen instances, even if RC is still the recommended course of treatment. According to this report, PC combined with lymphadenectomy and adjuvant radiation led to a five-year survival rate free of recurrence, indicating its possible therapeutic use.

## Conclusions

Localized SCC within bladder diverticula can be safely and effectively managed with PC combined with pelvic lymphadenectomy and adjuvant radiation therapy, especially in patients who are not candidates for RC. Because SCC is more aggressive and has a higher recurrence rate than UC, improving long-term outcomes requires early diagnosis, prompt management, and comprehensive follow-up.

Since adjuvant radiation greatly lowers the chance of local recurrence, it should be taken into consideration in situations involving extravesical involvement or pelvic lymph node involvement. However, despite the encouraging oncological control shown in this case, long-term prospective investigations are needed to provide definite therapy guidelines and refine patient selection criteria for bladder-preserving techniques in SCC care.
